# Painful Foot Lesions: A Case Report

**DOI:** 10.7759/cureus.33937

**Published:** 2023-01-18

**Authors:** Trevor J Lockard, Ritu N Swali, Melodi Javid Whitley

**Affiliations:** 1 Department of Dermatology, University of Nebraska Medical Center, Omaha, USA; 2 Department of Dermatology, Duke University, Durham, USA

**Keywords:** hand-foot skin reaction, dermatologic drug reaction, tyrosine kinase inhibitors, hyperkeratotic lesions, foot lesions, renal cell carcinoma

## Abstract

Hand-foot skin reaction (HFSR) is a documented cutaneous adverse reaction to tyrosine kinase inhibitor (TKI) chemotherapy. Cutaneous toxicities such as HFSR can be debilitating and may result in serious complications; however, continued chemotherapy is desirable to optimize the patient’s odds of survival and tumor remission. We present a case of a 66-year-old male, with a history of metastatic renal clear cell carcinoma, who was diagnosed with grade 3 HFSR triggered by axitinib, a tyrosine kinase inhibitor. Our patient was able to expeditiously resume chemotherapy after temporary cessation of axitinib with concurrent application of topical steroids and keratolytics. Expedient return to life-prolonging chemotherapy is of great importance for patients with advanced malignancies; therefore, accurate diagnosis and prompt identification of the offending medication are critical to the management of this entity. We aim to increase the awareness of tyrosine kinase inhibitor-induced HFSR and review the diagnosis and current guidelines for management.

## Introduction

Hand-foot skin reaction (HFSR) is a documented cutaneous adverse drug reaction caused by tyrosine kinase inhibitors (TKIs), such as axitinib [1]. This dermatologic adverse reaction to cancer treatment is of significance for practitioners of both dermatology and oncology. Cutaneous toxicities such as HFSR can be debilitating to the patient and may lead to serious complications that require treatment cessation; however, continued chemotherapy is desirable to optimize the patient’s odds of survival and tumor remission. Therefore, accurate diagnosis and prompt identification of the culprit medication are critical to the management of this entity. This article has been previously presented as an abstract and poster at the University of Nebraska Medical Center Graduate Medical Education Symposium on May 4, 2022, and as a poster at the 2022 Nebraska Dermatology Society Meeting on October 20, 2022.

## Case presentation

A 66-year-old male, with a history of metastatic renal clear cell carcinoma, presented to the dermatology clinic with a two-month history of painful lesions on the soles of his feet. These lesions were being repeatedly debrided by podiatry without improvement. The lesions had been worsening gradually over the past two weeks and now interfered with his activities of daily living. The patient had no other skin lesions or systemic symptoms such as weight loss, fevers, or arthralgias. He also denied environmental triggers or lifestyle changes, such as increased physical activity, that could otherwise induce callous formation or skin irritation. Of note, the patient had been taking axitinib 5 mg by mouth twice daily in combination with periodic infusions of 200 mg IV pembrolizumab for the management of metastatic renal clear cell carcinoma.

**Figure 1:**
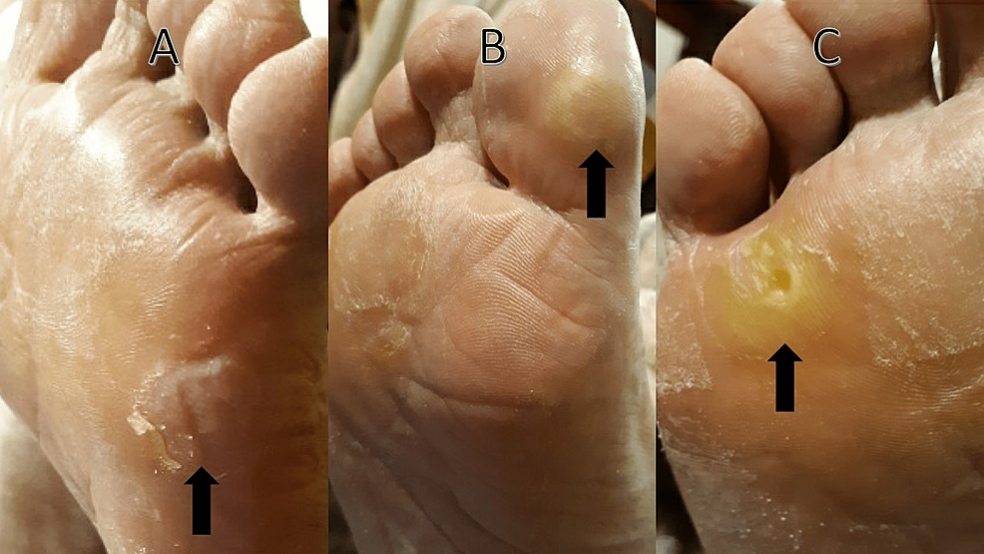
Hyperkeratotic desquamating plaques over the (A) left fifth metatarsal, (B) right great toe, and (C) right fourth metatarsophalangeal joint.

**Figure 2:**
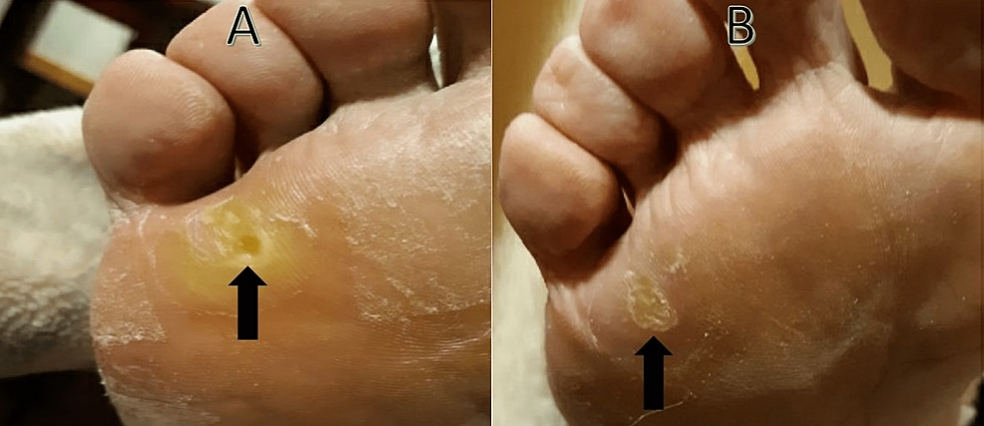
HFSR (A) before and (B) after treatment with topical keratolytics, topical steroids, and brief cessation of axitinib. HFSR: hand-foot skin reaction

On clinical examination, the patient had hyperkeratotic tender plaques with overlying superficial desquamation over the plantar surfaces of the right great toe, left fifth metatarsal, and bilateral fourth metatarsophalangeal joints (Figure 1A-1C). A diagnosis of hand-foot skin reaction (HFSR), presumably due to axitinib, was made. Based on the National Cancer Institute Common Terminology Criteria for Adverse Events (NCI CTCAE) and clinical practice guidelines, the reaction was graded at the maximum grade of 3, and axitinib was discontinued [2,3]. The patient was concurrently treated with topical urea 40% lotion and clobetasol 0.05% ointment twice daily. Given the prompt improvement in pain and lesion size within one week of cessation (Figure 2A-2B), the patient was successfully able to restart axitinib at a decreased dose of 3 mg twice daily instead of 5 mg twice daily. He has since tolerated axitinib therapy and no longer requires topical treatment for these lesions.

## Discussion

Hand-foot skin reaction is an uncommon drug reaction attributed to multikinase inhibitors, drugs that inhibit multiple receptor tyrosine kinases (RTKs). While the precise pathogenesis of HFSR is unknown, it is believed that the inhibition of RTKs, such as vascular endothelial growth factor receptors (VEGFR), particularly when combined with the inhibition of other endothelial RTKs, such as platelet-derived growth factor receptor (PDGFR), triggers the desquamation and hyperkeratosis observed in HFSR [1]. Friction and local irritation are implicated as drivers of the reaction. A meta-analysis of 57 studies tracking HFSR in VEGFR inhibitor TKIs showed the incidence of all-grade HFSR at 35% and grade 3 at 9.7% [4]. Further studies of HFSR incidence in TKIs that inhibited both PDGFR and VEGFR subtypes demonstrated dramatically increased incidence of HFSR; for example, the occurrence of all-grade HFSR was maximal at 65.2% with lenvatinib [5]. These data support the hypothesis that PDGFR and VEGFR co-inhibition is more likely to induce HFSR than the inhibition of either receptor alone.

The diagnosis of HFSR is clinical, requiring the presence of painful hyperkeratotic plaques with surrounding erythema in a patient currently taking a TKI. HFSR can be confused with the similarly named hand-foot syndrome, also called palmar-plantar erythrodysesthesia. However, hand-foot syndrome presents with more diffuse erythema with a preference for the palms and is traditionally found in patients taking cytotoxic chemotherapy rather than newer molecular agents (e.g., TKIs) [6]. The differentiation of these entities is particularly critical in patients taking combination chemotherapy regimens, as the diagnosis then determines which medication should be reduced or changed.

Axitinib is a second-generation tyrosine kinase inhibitor (TKI) that inhibits VEGFR-1, VEGFR-2, and VEGFR-3. It is indicated for the first-line treatment of advanced renal cell carcinoma combined with pembrolizumab or avelumab, but it can be used alone for treatment after failing at least one systemic therapy [7]. Concerning adverse effects of this drug include HFSR, hypertension, hepatotoxicity when used as combination therapy with pembrolizumab or avelumab, and impaired wound healing [7]. Axitinib lacks the PDGFR co-inhibition, which typically leads to high HFSR incidence, yet a significant proportion of patients develop HFSR while taking this drug. One meta-analysis estimates that all-grade HFSR incidence in patients taking axitinib is 29.2% and grade 3 incidence is 9.6% [8].

HFSR is graded per NCI CTCAE criteria (Table 1) for palmar-plantar erythrodysesthesia, as there are no criteria for HFSR separately. The patient’s presentation was consistent with grade 3 HFSR given his substantial limitations in activities of daily living [2]. The guidelines for the treatment of grade 3 HFSR are to stop the drug for a minimum of seven days or until the reaction grade is 0-1 and then resume at a decreased dose. Keratolytic agents (e.g., urea) and high-potency steroids (e.g., clobetasol) are also recommended [1,3]. Keratolytics provide relief by removing unwanted keratin from the epidermis and decreasing the friction to the areas. Superpotent steroids are effective anti-inflammatory agents in the thick skin of the palms and soles.

**Table 1:** National Cancer Institute Common Terminology Criteria for Adverse Events grading scale for palmar-plantar erythrodysesthesia and HFSR. *Skin changes include peeling, blisters, bleeding, fissures, edema, or hyperkeratosis. Adapted from the National Cancer Institute (2017) [2] HFSR: hand-foot skin reaction

Grade	Description
1	Minimal painless skin changes^*^ or dermatitis (e.g., erythema, edema, or hyperkeratosis)
2	Skin changes^*^ with pain, limiting instrumental activities of daily living
3	Severe skin changes^*^ with pain, limiting self-care activities of daily living

**Figure 3:**
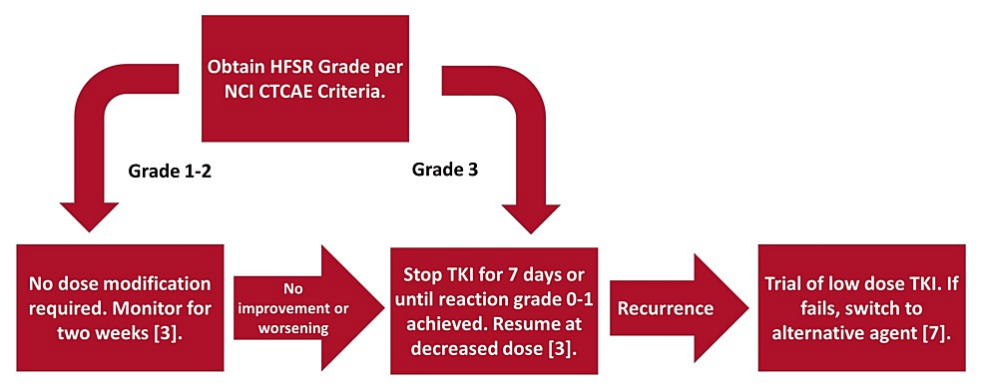
Algorithm for tyrosine kinase inhibitor (TKI) dose reduction in HFSR. Compiled from guidelines found in Lacouture et al. (2021) Source: [3,7] HFSR, hand-foot skin reaction; NCI CTCAE, National Cancer Institute Common Terminology Criteria for Adverse Events

Reaction grade and duration are the most important factors in the decision to reduce TKI dose. For grade 1 and 2 reactions, no dose changes are recommended unless the reaction fails to resolve within two weeks of initial recognition and therapy. For grade 3, however, prompt TKI withdrawal is indicated, with subsequent dose reduction per the manufacturer’s prescribing information [3]. The guidelines for TKI dose reduction are summarized in our decision-making algorithm (Figure 3). Providers must weigh the risks and benefits of the reduction or discontinuation of TKIs, as multiple studies have demonstrated the association between TKI-induced HFSR and overall tumor response (progression-free survival) in patients with solid tumors such as renal cell carcinoma and colon cancer [9,10]. Nakano et al. performed multivariate analysis of all TKI adverse reactions occurring in their study, as well as patient risk factors and laboratory values indicative of tumor severity. They correlated these variables with progression-free survival and found that of all these parameters, only the development of HFSR was predictive of progression-free survival in metastatic renal cell carcinoma treated with TKI therapy [9]. Therefore, it is strongly preferred to maintain therapy with the offending TKI for a long term, even if brief interruption is necessary.

## Conclusions

Expedient return to life-prolonging chemotherapy is critical for patients with advanced malignancies. When a high-grade HFSR diagnosis is made, providers should promptly execute a twofold management strategy of topical intervention and short-term drug interruption. Complex cutaneous adverse reactions such as HFSR warrant referral to dermatology for diagnosis and management to optimize patient outcomes. In this report, we aim to increase the awareness of tyrosine kinase inhibitor-induced hand-foot skin reaction among dermatologists and oncologists and review the current guidelines for management.
